# The IN-CARE Project: Socioeconomic Inequalities in Care Use and Provision Across Countries and Over Time

**DOI:** 10.1093/geroni/igaa057.2565

**Published:** 2020-12-16

**Authors:** Marjolein Broese van Groenou

## Abstract

Ageing societies and recent reforms to long-term care (LTC) in many European countries are likely to make informal care by kin and nonkin increasingly critical for fulfilling the care needs of older people. To date, it is unknown whether informal care falls disproportionately on disadvantaged populations. The IN-CARE project (a collaboration of Dutch, UK and German research teams; http://in-care.fk12.tu-dortmund.de/) examines if and how LTC reforms exacerbate existing social disparities in care use and provision in older age. To this end, this project compares the socioeconomic status (SES) gradient in formal and informal care across Europe and over time. A particular effort is made to include macro-level indicators of LTC systems in cross-level analyses across countries. The first paper presented in this symposium by the UK team studied SES-inequality in care receipt across European nations with different care systems; the second paper presented by the German team studied the same question but now among caregivers, the third paper provides the analyses for caregivers in Japan, and the fourth paper by the Dutch team studies SES-inequalities in care use within the Netherlands over time (1995-2015). The symposium will start off with a short description of the IN-CARE project (2019-2022). Tine Rostgaard agreed to be our discussant.

